# Non-survivor patients with malignant middle cerebral artery infarction showed persistently high serum malondialdehyde levels

**DOI:** 10.1186/s12883-019-1479-z

**Published:** 2019-10-17

**Authors:** Leonardo Lorente, María M. Martín, Pedro Abreu-González, Rafael Sabatel, Luis Ramos, Mónica Argueso, Jordi Solé-Violán, Juan J. Cáceres, Alejandro Jiménez, Victor García-Marín

**Affiliations:** 1 Intensive Care Unit, Hospital Universitario de Canarias, Ofra, s/n. La Laguna –, 38320 Santa Cruz de Tenerife, Spain; 2 Intensive Care Unit, Hospital Universitario Nuestra Señora de Candelaria, Crta del Rosario s/n, 38010 Santa Cruz de Tenerife, Spain; 3 Deparment of Phisiology, Faculty of Medicine, University of the La Laguna, 38320 Santa Cruz de Tenerife, La Laguna Spain; 4 Deparment of Radiology, Hospital Universitario de Canarias, Ofra, s/n. La Laguna –, 38320 Santa Cruz de Tenerife, Spain; 5 Intensive Care Unit, Hospital General La Palma, Buenavista de Arriba s/n, 38713 Breña Alta, La Palma Spain; 6 Intensive Care Unit, Hospital Clínico Universitario de Valencia, Avda. Blasco Ibáñez n°17-19, 46004 Valencia, Spain; 7 Intensive Care Unit, Hospital Universitario Dr. Negrín, CIBERES, Barranco de la Ballena s/n, 35010 Las Palmas de Gran Canaria, Spain; 8 Intensive Care Unit, Hospital Insular, Plaza Dr. Pasteur s/n, ia-35016 Las Palmas de Gran Canar, Spain; 9 Research Unit, Hospital Universitario de Canarias, Ofra, s/n. La Laguna –, 38320 Santa Cruz de Tenerife, Spain; 10 Deparment of Neurosurgery, Hospital Universitario de Canarias, Ofra, s/n. La Laguna –, 38320 Santa Cruz de Tenerife, Spain

**Keywords:** Malondialdehyde, Ischemic stroke, Patients, Mortality, Prognosis

## Abstract

**Objective:**

Previously there have been found higher circulating malondialdehyde levels during the first week of ischemic stroke in patients with worst neurological functional outcome, and at moment of ischemic stroke in non-survivor patients. Thus, the aim of our study was to determine the potential role of serum malondialdehyde levels during the first week of a severe cerebral infarction to mortality prediction.

**Methods:**

This study was observational, prospective, and multicenter. We included patients with a severe malignant middle cerebral artery infarction (MMCAI) defined as patients with computed tomography showing acute infarction in more than of 50% of the territory and Glasgow Coma Scale (GCS) lower than 9. We determined serum concentrations of malondialdehyde on days 1, 4 and 8 of MMCAI.

**Results:**

Serum malondialdehyde concentrations at days 1 (*p* < 0.001), 4 (*p* < 0.001), and 8 (*p* = 0.001) of MMCAI in non-survivor patients (*n* = 34) were higher than in survivor patients (*n* = 34). ROC curve analyses showed that serum malondialdehyde concentrations at days 1, 4, and 8 of MMCAI had an AUC (95% CI) to predict 30-day mortality of 0.77 (0.65–0.86; *p* < 0.001), 0.82 (0.69–0.91; *p* < 0.001) and 0.84 (0.70–0.93; *p* < 0.001) respectively.

**Conclusions:**

The new findings of our study were that serum malondialdehyde levels during the first week of MMCAI could be used as biomarkers to mortality prediction.

## Introduction

A large quantity of disabilities, deaths and resources consumption are generated by ischemic stroke [1]. In ischemic stroke, in addition to cell death produced by brain vasculature obstruction that causes a reduction of blood containing oxygen and substrates to neurons, could appears a secondary brain injury mediated by oxidative stress [2–6]. Different end-products could appear during lipid peroxidation such as malondialdehyde, which is formed during cellular membrane phospholipids degradation [3, 4]. Afterwards malondialdehyde could be released to extracellular space and appears in the blood; and circulating malondialdehyde levels have been used as lipid oxidation biomarker [7, 8].

Previously have been found higher circulating malondialdehyde levels during the first week of ischemic stroke in patients with worst neurological functional outcome [9–12], and at moment of ischemic stroke in non-survivor patients [13, 14]. Thus, the aim of our study was to determine the potential role of serum malondialdehyde levels during the first week of a severe cerebral infarction to mortality prediction.

## Methods

### Design and subjects

This study was observational and prospective. This multicentre study was performed with the Institutional Review Board approval of the six participating hospitals and with the written informed consent of patient legal guardians. This study was carried out in the Intensive Care Units of the following hospitals: H. General de La Palma from Breña Alta, H. Universitario Dr. Negrín from Las Palmas de Gran Canaria, H. Insular from Las Palmas de Gran Canaria, H. Universitario Nuestra Señora de Candelaria from Santa Cruz de Tenerife, H. Clínico Universitario de Valencia from Valencia, and H. Universitario de Canarias from La Laguna.

We included patients with a severe malignant middle cerebral artery infarction (MMCAI), defined as computed tomography showing acute infarction in more than of 50% of the territory and Glasgow Coma Scale (GCS) [15] lower than 9; and there were excluded patients with brain hemorrhage, less than 18 years of age, inflammatory or malignant disease, or pregnancy.

Previously, our team determined serum malondialdehyde concentrations in some of those patients in the day of a severe MMCAI [14]. In this current work, we determine serum malondialdehyde concentrations at days 1, and also at days 4 and 8.

### Clinical and demographic variables

We recorded the following variables from the patients: age, arterial hypertension, sex, chronic obstructive pulmonary disease (COPD), diabetes mellitus, heart failure, chronic renal failure, temperature, CGS, lactic acid, sodium, bilirubin, creatinine, glycaemia, pressure of arterial oxygen (PaO_2_), fraction inspired oxygen (FI0_2_), PaO_2_/FIO_2_ ratio, platelets, leukocytes, haemoglobin, fibrinogen, international normalized ratio (INR), activated partial thromboplastin time (aPTT), Acute Physiology and Chronic Health Evaluation II (APACHE II) score [16], thrombolysis, volumen infarction, haemorrhagic transformation, midline shift, and descompresive craniectomy. Thirty-day mortality was considered as the end-point study.

### Meassure of serum malondialdehyde concentrations

We obtained serum blood samples on days 1, 4 and 8 of MMCAI and were frozen at − 80 °C until the determination of serum malondialdehyde concentrations. All assays for the meassure of malondialdehyde concentrations were carried out in the Physiology Department of Medicine Faculty of La Laguna University (Tenerife, Spain). The meassure of malondialdehyde concentrations was performed according to thiobarbituric acid-reactive substance (TBARS) method by Kikugawa et al. [17]. We mixed serum (200 μL), thiobarbituric acid (2.5 mL at 0.8%), sodium dodecyl sulfate (200 μL at 8.1%), trichloroacetic acid (1.5 mL with pH 3.5) and butylated hydroxytoluene (50 μL at 0.8%). We kept the mixture during 1 h at 5 °C and later it was heated during 1 h at 100 °C. Afterwards, n-butanol was extracted. Finally, the sample was placed doubly in a 96-well plate and read at 535 nm with a spectrophotometer reader (Benchmark Plus, Bio-Rad, Hercules, CA, USA). The assay detection limit, intra-assay coefficient variation, and inter-assay coefficient variation were of 0.08 nmol/ml, 1.82, and 4.01% respectively.

### Statistical methods

Medians (and interquartile ranges) were used to describe continuous variables, and frequencies (and percentages) to describe categorical variables. Wilcoxon-Mann-Whitney test was used to compare continuous variables between survivor and non-survivor patient groups, and chi-square test to compare categorical variables between patient groups. Receiver operating characteristic (ROC) analyses were used to determine the capacity for 30-day mortality prediction by serum malondialdehyde levels at day 1, 4 and 8 of MMCAI. Area under curve (AUC), and sensitivity, specificity, positive likelihood ratio, negative likelihood ratio, positive predicted value and negative predicted value of serum malondialdehyde levels cut-offs for mortality prediction are showed with its 95% confidence intervals (CI). Optimal cut-off values at days 1, 4 and 8 were selected according to Youden J index. Multiple logistic regression was carried out to determine whether exists an association between serum malondialdehyde levels and 30-day mortality after to control for platelet count, lactic acid and GCS. *P-*values< 0.05 was the point considered to determine as statistically significant. SPSS 17.0 (SPSS Inc., Chicago, IL, USA), LogXact 4.1 (Cytel Co., Cambridge, MA), and NCSS 2000 (Kaysville, Utah) were the programs used for statistical analyses.

## Results

In Table 1 is possible to see that significant differences were not found between patients groups, non-survivors (*n* = 34) and non-survivors (n = 34), in age, arterial hypertension, sex, COPD, diabetes mellitus, heart failure, chronic renal failure, temperature, lactic acid, sodium, bilirubin, creatinine, glycaemia, PaO_2_, PaO_2_/FIO_2_ ratio, leukocytes, haemoglobin, fibrinogen, INR, aPTT, APACHE-II, thrombolysis, volumen infarction, haemorrhagic transformation, midline shift, and descompresive craniectomy. However, lower GCS and platelets were found in non-survivor than in survivor patients.

**Table 1:** Clinical and biochemical characteristics of 30-day survivor and non-survivor MMCAI patients

	Non-survivors (*n* = 34)	Survivors (*n* = 34)	*P*-value
Age (years) - median (p 25–75)	63 (53–70)	59 (47–68)	0.36
Arterial hypertension - n (%)	16 (47.1)	19 (55.9)	0.63
Gender female - n (%)	13 (38.2)	14 (41.2)	0.99
COPD - n (%)	1 (2.9)	1 (2.9)	0.99
Diabetes mellitus - n (%)	9 (26.5)	4 (11.8)	0.22
Heart failure - n (%)	1 (2.9)	1 (2.9)	0.99
Chronic renal failure - n (%)	2 (5.9)	2 (5.9)	0.99
Temperature (°C) - median (p 25–75)	36.9 (36.0–37.3)	36.4 (36.0–37.0)	0.15
GCS score - median (p 25–75)	6 (3–7)	7 (6–8)	0.01
Lactic acid (mmol/L)-median (p 25–75)	1.55 (1.00–2.70)	1.20 (0.90–1.70)	0.05
Sodium (mEq/L)- median (p 25–75)	140 (139–145)	139 (136–145)	0.38
Bilirubin (mg/dl) - median (p 25–75)	0.60 (0.33–1.10)	0.60 (0.40–0.83)	0.95
Creatinine (mg/dl) - median (p 25–75)	1.00 (0.70–1.25)	0.80 (0.60–1.13)	0.19
Glycemia (g/dL) - median (p 25–75)	136 (118–162)	127 (100–170)	0.40
PaO2 (mmHg) - median (p 25–75)	115 (94–267)	156 (105–293)	0.26
PaO2/FI0_2_ ratio - median (p 25–75)	254 (192–325)	300 (198–369)	0.24
Platelets - median*10^3^/mm^3^ (p 25–75)	175 (136–216)	202 (171–265)	0.02
Leukocytes-median*10^3^/mm^3^ (p 25–75)	13.9 (9.7–20.1)	12.4 (9.6–16.9)	0.32
Hemoglobin (g/dL) - median (p 25–75)	12.5 (11.0–14.8)	12.1 (11.4–14.0)	0.81
Fibrinogen (mg/dl) - median (p 25–75)	419 (337–631)	443 (416–489)	0.90
INR - median (p 25–75)	1.20 (1.01–1.31)	1.06 (1.00–1.20)	0.07
aPTT (seconds) - median (p 25–75)	27 (26–32)	28 (25–30)	0.91
APACHE-II score - median (p 25–75)	22 (19–27)	20 (16–25)	0.06
Thrombolysis - n (%)	10 (29.4)	11 (32.4)	0.99
Volumen infarction (ml) - median (p25–75)	180 (60–277)	173 (100–231)	0.64
Haemorrhagic transformation - n (%)	6 (17.6)	7 (20.6)	0.99
Midline shift (mm) - median (p 25–75)	9.0 (3.5–15.0)	6.0 (2.5–11.5)	0.43
Decompressive craniectomy – n (%)	7 (20.6)	9 (26.5)	0.78
Malondialdehyde (nmol/mL)-median (p 25–75)	2.96 (2.01–4.44)	1.84 (1.33–2.28)	< 0.001

*P 25–75* percentile 25th–75th, *COPD* chronic obstructive pulmonary disease, *GCS* Glasgow Coma Scale, *PaO*_*2*_ pressure of arterial oxygen/fraction inspired oxygen, *FIO*_*2*_ fraction inspired oxygen, *INR* international normalized ratio, *aPTT* activated partial thromboplastin time, *APACHE II* Acute Physiology and Chronic Health Evaluation

**Fig. 1:**
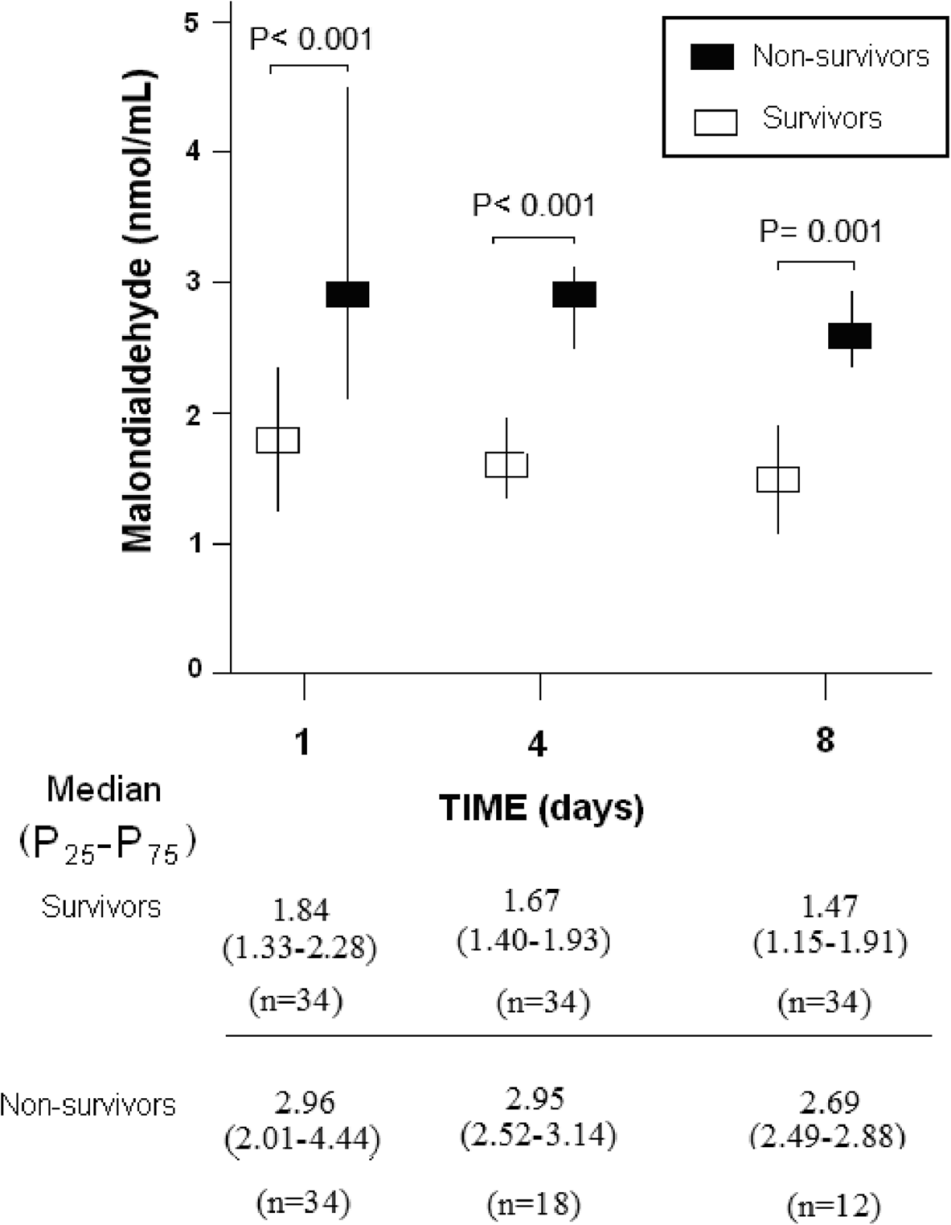
Serum malondialdehyde levels at day 1, 4 and 8 of severe malignant middle cerebral artery infarction in 30-day survivor and non-survivor patients

Serum MDA concentrations at days 1 (*p* < 0.001), 4 (*p* < 0.001), and 8 (*p* = 0.001) of MMCAI in non-survivor patients were higher than in survivor patients (Table 2 and Fig. 1).

**Table 2:** Receiver operation characteristic analysis using serum malondialdehyde levels at day 1, 4 and 8 of severe malignant middle cerebral artery infarction as predictor of mortality at 30 days

	Day 1	Day 4	Day 8
Cut-off of malondialdehyde (mmol/mL)	> 2.87	> 2.12	> 2.01
Sensitivity and 95% CI	58.8 (40.7–75.4)	83.3 (58.6–96.4)	83.3 (51.6–97.9)
Specificity and 95% CI	94.1 (80.3–99.3)	91.2 (76.3–98.1)	94.1 (80.3–99.3)
Positive likelihood ratio and 95% CI	10.0 (2.5–39.5)	9.4 (3.1–28.4)	14.2 (3.6–55.6)
Negative likelihood ratio and 95% CI	0.4 (0.3–0.7)	0.2 (0.1–0.5)	0.2 (0.1–0.6)
Positive predicted value and 95% CI	90.9 (71.7–97.5)	83.3 (62.5–93.8)	83.3 (56.0–95.2)
Negative predicted value and 95% CI	69.6 (60.3–77.5)	91.2 (78.5–96.7)	94.1 (81.8–98.3)

*CI* confidence intervals

ROC curve analyses showed that serum malondialdehyde concentrations at days 1, 4, and 8 of MMCAI had an AUC (95% CI) to predict 30-day mortality of 0.77 (0.65–0.86; *p* < 0.001), 0.82 (0.69–0.91; *p* < 0.001) and 0.84 (0.70–0.93; *p* < 0.001) respectively. Table 2 showed sensitivity, specificity, positive likelihood ratio, negative likelihood ratio, positive predicted value and negative predicted value of serum malondialdehyde levels cut-offs at days 1, 4, and 8 of MMCAI for mortality prediction.

Multiple logistic regression an association between serum malondialdehyde levels> 2.87 mmol/mL and 30-day mortality (OR = 6.509; 95% CI = 2.095–20.222; *p* = 0.001) after to control for platelet count, lactic acid and GCS (Table 3).

**Table 3:** Multiple logistic regression analysis to predict 30-day mortality

Variable	Odds Ratio	95% Confidence Interval	*P*-value
Platelet count (each 1000/mm^3^)	0.995	0.985–1.006	0.39
Lactic acid (mmol/L)	0.967	0.486–1.925	0.92
Glasgow Coma Scale (points)	0.528	0.336–0.829	0.01
Serum malondialdehyde > 2.87 mmol/mL	6.509	2.095–20.222	0.001

## Discussion

The new findings of our study were that serum malondialdehyde levels during the first week of MMCAI could be used as biomarkers to mortality prediction.

Previously have been found higher circulating malondialdehyde levels during the first week of ischemic stroke in patients with worst neurological functional outcome [9–12], and at moment of ischemic stroke in non-survivor patients [13, 14]. Thus, the higher serum MDA levels during the first week of MMCAI observed in non-survivor patients in respect to survivor patients, and that those levels could be used as mortality prediction are two novel findings of our study.

We believed that those higher concentrations of serum malondialdehyde during the first week of MMCAI in non-survivor patients reflects a higher ROS production and lipid peroxidation in comparison to survivor patients, and the use of antioxidant agents could be a new therapeutic to explore in MMCAI patients. The administration of melatonin in animal models of ischemic stroke has been associated with a reduction of oxidation [18–22], specifically a reduction of malondialdehyde levels [21, 22], and even an increase of survival. In patients with ischemic stroke, the oral administration of different antioxidant vitamins (B2, B6, B12, C, E) during the first 14 days of stroke has been associated with lower plasma levels of malondialdehyde [23–25]. Thus, although we recognize that our study has the limitation that other oxidant state compounds were not reported, we think that all those findings could open the interest for study in patients with ischemic strike the oxidative stress and the potential role of antioxidant agents in your treatment.

## Conclusions

The new findings of our study were that serum malondialdehyde levels during the first week of MMCAI could be used as biomarkers to mortality prediction.

## Data Availability

The datasets used and/or analysed during the current study are available from the corresponding author on reasonable request.

## Abbreviations

APACHE: Acute Physiology and Chronic Health Evaluation
aPTT: activated partial thromboplastin time
COPD: Chronic Obstructive Pulmonary Disease
FIO_2_: fraction inspired of oxygen
GCS: Glasgow Coma Scale
ICU: Intensive Care Unit
INR: International normalized ratio
PaO_2_: Pressure of arterial oxygen/fraction inspired oxygen

## References

[1] Adams HP, del Zoppo G, Alberts MJ, Bhatt DL, Brass L, Furlan A, Grubb RL, Higashida RT, Jauch EC, Kidwell C, Lyden PD, Morgenstern LB, Qureshi AI, Rosenwasser RH, Scott PA, Wijdicks EF (2007). American Heart Association; American Stroke Association Stroke Council; Clinical Cardiology Council; Cardiovascular Radiology and Intervention Council; Atherosclerotic Peripheral Vascular Disease and Quality of Care Outcomes in Research Interdisciplinary Working Groups. Guidelines for the early management of adults with ischemic stroke: a guideline from the American Heart Association/American Stroke Association Stroke Council, Clinical Cardiology Council, Cardiovascular Radiology and Intervention Council, and the Atherosclerotic Peripheral Vascular Disease and Quality of Care Outcomes in Research Interdisciplinary Working Groups: the American Academy of Neurology affirms the value of this guideline as an educational tool for neurologists. Stroke.

[2] Radak D, Resanovic I, Isenovic ER (2014). Link between oxidative stress and acute brain ischemia. Angiology.

[3] Rodrigo R, Fernández-Gajardo R, Gutiérrez R, Matamala JM, Carrasco R, Miranda-Merchak A, Feuerhake W (2013). Oxidative stress and pathophysiology of ischemic stroke: novel therapeutic opportunities. CNS Neurol Disord Drug Targets.

[4] Manzanero S, Santro T, Arumugam TV (2013). Neuronal oxidative stress in acute ischemic stroke: sources and contribution to cell injury. Neurochem Int.

[5] Pradeep H, Diya JB, Shashikumar S, Rajanikant GK (2012). Oxidative stress--assassin behind the ischemic stroke. Folia Neuropathol.

[6] Warner DS, Sheng H, Batinić-Haberle I (2004). Oxidants, antioxidants and the ischemic brain. J Exp Biol.

[7] Draper HH, Hadley M (1990). Malondialdehyde determination as index of lipid peroxidation. Methods Enzymol.

[8] Dalle-Donne I, Rossi R, Colombo R, Giustarini D, Milzani A (2006). Biomarkers of oxidative damage in human disease. Clin Chem.

[9] Ozkul A, Akyol A, Yenisey C, Arpaci E, Kiylioglu N, Tataroglu C (2007). Oxidative stress in acute ischemic stroke. J Clin Neurosci.

[10] Polidori MC, Cherubini A, Stahl W, Senin U, Sies H, Mecocci P (2002). Plasma carotenoid and malondialdehyde levels in ischemic stroke patients: relationship to early outcome. Free Radic Res.

[11] Silina EV, Rumiantseva SA, Bolevich SB, Men'shova NI (2011). Course of free radical processes and prognosis of ischemic and hemorrhagic stroke. Zh Nevrol Psikhiatr Im S S Korsakova.

[12] Tsai NW, Chang YT, Huang CR, Lin YJ, Lin WC, Cheng BC, Su CM, Chiang YF, Chen SF, Huang CC, Chang WN, Lu CH (2014). Association between oxidative stress and outcome in different subtypes of acute ischemic stroke. Biomed Res Int.

[13] Kotur-Stevuljevic J, Bogavac-Stanojevic N, Jelic-Ivanovic Z, Stefanovic A, Gojkovic T, Joksic J, Sopic M, Gulan B, Janac J, Milosevic S (2015). Oxidative stress and paraoxonase 1 status in acute ischemic stroke patients. Atherosclerosis.

[14] Lorente L, Martín MM, Abreu-González P, Ramos L, Argueso M, Solé-Violán J, Riaño-Ruiz M, Jiménez A (2015). Serum malondialdehyde levels in patients with malignant middle cerebral artery infarction are associated with mortality. PLoS One.

[15] Teasdale G, Jennett B (1974). Assessement of coma and impaired conciousness. A practical scale. Lancet.

[16] Knaus WA, Draper EA, Wagner DP, Zimmerman JE (1985). APACHE II: a severity of disease classification system. Crit Care Med.

[17] Kikugawa K, Kojima T, Yamaki S, Kosugi H (1992). Interpretation of the thiobarbituric acid reactivity of rat liver and brain homogenates in the presence of ferric ion and ethylediaminotetraacetic acid. Anal Biochem.

[18] Tai SH, Hung YC, Lee EJ, Lee AC, Chen TY, Shen CC, Chen HY, Lee MY, Huang SY, Wu TS (2011). Melatonin protects against transient focal cerebral ischemia in both reproductively active and estrogen-deficient female rats: the impact of circulating estrogen on its hormetic dose-response. J Pineal Res.

[19] Li H, Wang Y, Feng D, Liu Y, Xu M, Gao A, Tian F, Zhang L, Cui Y, Wang Z, Chen G (2014). Alterations in the time course of expression of the Nox family in the brain in a rat experimental cerebral ischemia and reperfusion model: effects of melatonin. J Pineal Res.

[20] Bhattacharya P, Pandey AK, Paul S, Patnaik R (2014). Melatonin renders by protein kinase C mediated aquaporin-4 inhibition in animal model of focal cerebral ischemia. Life Sci.

[21] Balduini W, Carloni S, Perrone S, Bertrando S, Tataranno ML, Negro S, Proietti F, Longini M, Buonocore G (2012). The use of melatonin in hypoxic-ischemic brain damage: an experimental study. J Matern Fetal Neonatal Med.

[22] Cuzzocrea S, Costantino G, Gitto E, Mazzon E, Fulia F, Serraino I, Cordaro S, Barberi I, De Sarro A, Caputi AP (2000). Protective effects of melatonin in ischemic brain injury. J Pineal Res.

[23] Ullegaddi R, Powers HJ, Gariballa SE (2004). B-group vitamin supplementation mitigates oxidative damage after acute ischaemic stroke. Clin Sci (Lond).

[24] Ullegaddi R, Powers HJ, Gariballa SE (2005). Antioxidant supplementation enhances antioxidant capacity and mitigates oxidative damage following acute ischaemic stroke. Eur J Clin Nutr.

[25] Ullegaddi R, Powers HJ, Gariballa SE (2006). Antioxidant supplementation with or without B-group vitamins after acute ischemic stroke: a randomized controlled trial. JPEN J Parenter Enteral Nutr.

